# Chemotherapy Related Cardiotoxicity Evaluation—A Contemporary Review with a Focus on Cardiac Imaging

**DOI:** 10.3390/jcm13133714

**Published:** 2024-06-26

**Authors:** Isabel G. Scalia, Bashaer Gheyath, Balaji K. Tamarappoo, Rohit Moudgil, James Otton, Milagros Pereyra, Hema Narayanasamy, Carolyn Larsen, Joerg Herrmann, Reza Arsanjani, Chadi Ayoub

**Affiliations:** 1 Department of Cardiovascular Medicine, Mayo Clinic, Phoenix, AZ 85054, USA; scalia.isabel@mayo.edu (I.G.S.);; 2 Department of Imaging, Cedars Sinai Medical Center, Los Angeles, CA 90048, USA; 3 Division of Cardiology, Banner University Medical Center, The University of Arizona College of Medicine, Phoenix, AZ 85004, USA; 4 Department of Cardiology, Heart and Vascular Institute, Cleveland Clinic, Cleveland, OH 44195, USA; 5 Clinical School, St. Vincent’s Hospital, UNSW, Sydney, NSW 2010, Australia; 6 Department of Cardiovascular Medicine, Mayo Clinic, Rochester, MN 55905, USA

**Keywords:** cardiotoxicity, cardio-oncology, chemotherapy, echocardiography, cardiac imaging

## Abstract

The long-term survivorship of patients diagnosed with cancer has improved due to accelerated detection and rapidly evolving cancer treatment strategies. As such, the evaluation and management of cancer therapy related complications has become increasingly important, including cardiovascular complications. These have been captured under the umbrella term “cardiotoxicity” and include left ventricular dysfunction and heart failure, acute coronary syndromes, valvular abnormalities, pericardial disease, arrhythmia, myocarditis, and vascular complications. These complications add to the burden of cardiovascular disease (CVD) or are risk factors patients with cancer treatment are presenting with. Of note, both pre- and newly developing CVD is of prognostic significance, not only from a cardiovascular perspective but also overall, potentially impacting the level of cancer therapy that is possible. Currently, there are varying recommendations and practices regarding CVD risk assessment and mitigating strategies throughout the cancer continuum. This article provides an overview on this topic, in particular, the role of cardiac imaging in the care of the patient with cancer. Furthermore, it summarizes the current evidence on the spectrum, prevention, and management of chemotherapy-related adverse cardiac effects.

## 1. Key Points

Advances in cancer therapies have expanded the possibilities and survival of cancer patients. This now exposes patients at higher baseline risk for complications, such as those with pre-existing cardiovascular disease (CVD) and generates the risk of new CVDs as a consequence of the cancer therapies, grouped as “cardiotoxicity”. 

Baseline assessment, including the patient’s cardiovascular risk profile, as well as consequences associated with specific antineoplastic treatment, needs to be taken into consideration prior to the commencement of therapy. This may allow for primary preventative therapy to minimize cardiac risk, as well as guide clinicians toward the more appropriate modalities and frequency for the monitoring of cardiotoxicity. 

Cardiac imaging plays an important role in the early detection and evaluation of cardiotoxicity, allowing for effective treatment strategies that may further improve prognosis.

## 2. Introduction

Advances in early cancer detection and effective cancer treatment have led to a marked improvement in long-term survival rates [1,2]. Additionally, with the aging of the population and improved screening and diagnostic modalities, there has been a steady increase in the incidence of cancer over recent decades [3]. By 2040, estimations suggest a 47% increase in new cancer diagnoses worldwide [4]. The rapidly evolving range of novel cancer therapies and the associated improved mortality has its own challenges, emphasizing the importance of healthy survivorship [2,5]. 

Cardiovascular disease (CVD) is one of the leading causes of death among important subgroups of cancer survivors [6,7,8]. Recent studies add to the growing knowledge that some of the highest cardiovascular risk dynamics are observed within the first year of cancer diagnosis [9]. This may relate to the multiple potentially cardiotoxic chemotherapy or immunotherapy agents that cancer patients receive [5,10]. Furthermore, there is an increasing number of patients with pre-existing CVD or cardiovascular risk factors, which may increase the risk of cancer therapy related cardiotoxicity, including left ventricular systolic dysfunction (LVSD), diastolic dysfunction and heart failure, myocarditis, acute coronary syndromes (ACS), arterial hypertension, dyslipidemia, arterial and venous thromboembolic events, valvular heart disease, pulmonary hypertension and arrhythmias including atrial fibrillation, QT prolongation related ventricular arrhythmia, and high-grade conduction disease [6,7,11,12,13,14,15].

**Figure 1:**
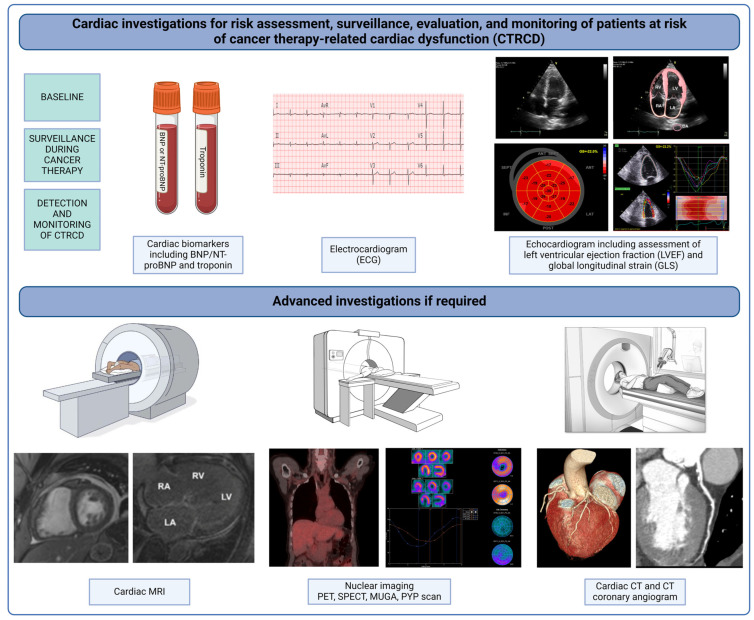
Graphical depiction of cardiac investigations involved in the baseline assessment, surveillance, and monitoring of cancer patients at risk of cancer therapy-related cardiac dysfunction. BNP, brain natriuretic peptide; NT-proBNP, N-terminal-proBNP; MRI, magnetic resonance imaging; PET, positron emission tomography; SPECT, single-photon emission computed tomography; MUGA, multigated acquisition scan; PYP, technetium-99 m pyrophosphate scintigraphy; CT, computed tomography.

Cardiotoxicity, also referred to as cancer therapy-related cardiac dysfunction (CTRCD) [8] or cancer therapy related CVD (CTR-CVD) [15]. It can have significant deleterious effects on patients, including increased morbidity and burden of medical interactions, reduction in quality of life, and the potential for disruption of cancer treatments [15]. Early detection is critical, especially in patients who are at increased risk of cardiotoxicity. Importantly, asymptomatic presentations are more common and are said to precede signs and symptoms of disease [14]. Clinically, cardiac investigations including, biomarkers and imaging, play a central role in surveillance for cardiotoxicity in cancer patients (Figure 1) [8].

There are numerous guidelines regarding risk assessment, diagnostic evaluation, and management of cardiotoxicity [6,7,12,13,15]. Overall, a collaborative multidisciplinary approach and a broad understanding of the strengths and weaknesses of current cardiac imaging techniques are essential [7]. This article reviews the current literature regarding chemotherapy induced cardiotoxicity, with a focus on the prevention, early detection, and management of left ventricular dysfunction. 

## 3. Cancer Therapy-Related Cardiac Dysfunction (CTRCD)

CTRCD or cardiotoxicity encompasses a wide spectrum of acute and sub-acute adverse cardiac effects from cancer treatments, including pharmacological treatment, immunotherapy, and radiation therapy [7,12,13]. LVSD is the most frequently encountered manifestation of CTRCD in cardio-oncology clinics [8,14,16]. Expert-consensus clinical guidelines broadly agree on the definition and grading of CTRCD related LVSD, as summarized in Table 1 [7,8,15]. It can range from a mild asymptomatic decline in left ventricular systolic function or elevation of cardiac biomarkers to symptomatic heart failure.

In asymptomatic patients, diagnosis and grading is typically based on echocardiographic assessment. Mild severity is considered for any patient with normal left ventricular systolic function at baseline, as measured by a left ventricular ejection fraction (LVEF) of ≥50%, with a new decline in global longitudinal strain (GLS) [reduction by >15% from prior] or a new elevation of cardiac biomarkers [15]. Moderate LVSD is considered as a ≥10% reduction of LVEF from baseline or a new reduction in LVEF < 10% to an LVEF 40–49% with a concurrent decline in GLS > 15% from baseline or rise in cardiac biomarkers [2,5,15]. 

Severe LVSD is considered in any patient with a new LVEF <40% per the European Society of Cardiology (ESC) 2022 guidelines, despite LVEF being considered as severely impaired by echocardiographic criteria when <30% [15]. LVSD can also be diagnosed symptomatically as a new onset of heart failure symptoms with supportive LVEF reduction or diagnostic biomarkers [14]. Despite these cutoffs, a decline in LVEF to <50% has been shown to be associated with poor overall prognosis and increased risk of cardiovascular events, especially when LVSD persists [14,17]. Clinically, due to the inter-technique variability of LVEF measurements, it is important that the same modality and technique be used for serial LVEF assessment where possible [7,12]. 

The severity and the reversibility of CTRCD are variable and are often dependent on both patient and cancer therapy-related factors [15]. CTRCD classification by reversibility patterns has previously been proposed, with type I (dose-related myocyte injury and thought to be irreversible) and type II injuries (shown to have reversibility with drug cessation); however, with complex regimens and sometimes a brief administration of agents, this classification may be difficult to apply in clinical practice and is less used nowadays [12,18]. Furthermore, CTRCD is a dynamic process and, therefore, may be acute or subacute, around the time of cancer therapy, or may occur as a delayed or chronic manifestation well after the cessation or completion of therapy [13,15]. 

## 4. Risk Assessment

**Figure 2:**
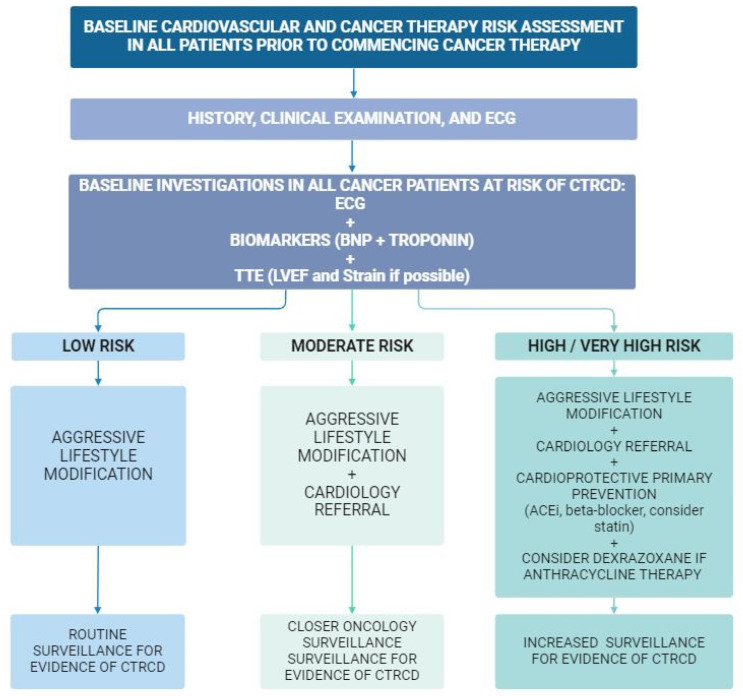
Flow chart of suggested baseline risk assessment for all cancer patients prior to receiving potentially cardiotoxic cancer therapy. Surveillance for cancer therapy-related cardiac dysfunction (CTRCD) is suggested based on stratification of baseline risk. This figure has been adapted from the 2022 ESC Guidelines on cardio-oncology [15]. ECG, electrocardiogram; BNP, brain natriuretic peptide; TTE, transthoracic echocardiogram; LVEF, left ventricular ejection fraction; ACEi, angiotensin-converting enzyme inhibitor.

A CVD risk assessment to assess for the potential for CTRCD is recommended for all patients prior to the commencement of cancer therapy [15,19]. This risk assessment is to take into consideration both cancer therapy related risk and patient-specific risk factors, electrocardiogram (ECG) and, where indicated, be complemented by a baseline cardiovascular evaluation including imaging and blood biomarkers (Figure 2).

Patient-related risk factors include the presence of baseline CVD, such as heart failure, valvular heart disease, arrhythmia, and pulmonary hypertension, underlying cardiovascular risk factors such as smoking, diabetes, hypertension, and obesity, genetic predisposition to CVD and toxicities, and comorbidities such as chronic kidney disease, as well as prior cancer treatments such as mediastinal radiation therapy or anthracycline chemotherapy (Table 2) [15,19,20].

Cancer therapy-related risk factors include the known cardiotoxic effects of the specific cancer therapy, both in terms of frequency and severity, and the use of multiple therapies, such as the combination of anthracycline therapy with targeted therapy, such as trastuzumab, or mediastinal radiation therapy (Table 3) [19]. Importantly, CTRCD risk is dynamic and risk assessment should occur both at baseline, to guide the frequency of surveillance intensity of prevention efforts, and again throughout and after cancer treatments [6]. 

### 4.1. Patient-Related Factors for CTRCD

Increasing research has been directed toward the development of clinical risk calculators that may provide a more quantitative CTRCD risk assessment [14,15]. For example, a high (>4 points) Systemic Coronary Risk Estimation (SCORE), developed by The European Society of Cardiology in the general population, has been associated with a significantly increased risk of CTRCD in the cancer population [23]. However, the SCORE model has also been found to overestimate cardiovascular risk in patients over 65 years old in general [20]. Modified models including SCORE2 and SCORE2-older persons have been recommended for all cancer patients prior to the commencement of therapy as a general CVD risk assessment [15,24]. Some cardio-oncology specific risk scores have been proposed, such as with human epidermal growth factor receptor-2 (HER2) targeted therapy. However, variations in the definition of CTRCD have made comparisons difficult, and predicting HER2 CTRCD risk with the proposed scores may not be precise [25]. 

At present, such risk scores should be combined with clinical evaluation, including a thorough medical history, physical examination, baseline blood tests, and ECG, prior to the initiation of any cancer therapy [6,7,10,12,15,26]. Patients at higher risk for CTRCD have been recommended further evaluation with cardiac imaging, typically comprehensive transthoracic echocardiography, and subsequent cardio-oncology or cardiology referral as part of the pre-chemotherapy evaluation [5,7,15]. Newer ESC guidelines and the American Heart Association (AHA) statement recommend baseline transthoracic echocardiogram (TTE) for many classes of chemotherapeutic agents regardless of patients’ calculated baseline risk [15,27]. 

### 4.2. Cardiac-Specific Biomarkers

Cardiac biomarkers may also be useful for risk stratification, surveillance, early diagnosis, and prognostication of CTRCD [28]. The most utilized and widely available biomarkers currently are cardiac specific troponins and brain natriuretic peptide (BNP) or N-terminal prohormone-BNP (NT-proBNP). The prognostic value of baseline serum biomarker measurements prior to antineoplastic therapy is unclear, with conflicting results from several studies [29,30,31]. However, there is ongoing promise for the clinical utility of serial biomarker measurements for the detection of early CTRCD, which will be reviewed in greater detail in the surveillance during therapy section below [6,12,32]. While baseline cardiac markers may be useful as a baseline, for comparison, in the event that the patient becomes symptomatic in the future, they may also present challenges to patient care; for example, mild asymptomatic elevation of troponin may lead to delays in cancer therapy while further cardiac evaluation is obtained.

### 4.3. Cancer Treatment-Related Risk Factors for CTRCD

Cardiotoxicity is a recognized adverse outcome in many chemotherapeutics; however, the risk is greater in several classes. A summary of the more common agents associated with LVSD is shown in Table 4. Over the last decade, there has been a rapid and substantial increase in the number of new cancer therapy agents clinically available, and as such, their cardiovascular side effects are becoming increasingly understood [14,33]. Furthermore, in some instances, LVSD has been reported as an adverse effect only after the chemotherapy agent has made it to the market, and as such, awareness of risk with their use may be limited [6,7,13]. The 2022 ESC guidelines outline cancer therapy-specific risk proformas in agreement with a previous document by the International Cardio-Oncology Society [14,19]. The value of these therapy-specific risk assessments is being evaluated currently. Some studies indicate that this approach may fare better than others. However, it may not identify patients at low risk of CTRCD who may not need intensive monitoring and primary prevention efforts.

Anthracyclines are the most common chemotherapeutic drug class to cause LVSD, with this phenomenon first described in cancer patients in 1979 [34]. Anthracyclines are used in the treatment of some lymphomas, leukemias, sarcomas, and breast cancers. Anthracycline and alkylating agents have been shown to have a dose-related risk for LVSD [15]. Doses of doxorubicin ≥250 mg/m^2^, epirubicin ≥600 mg/m^2^, and cyclophosphamide doses of ≥1.5 g/m^2^ are associated with higher risk for LVSD, with risk being proportionally higher with increasing doses [6,13,35,36]. However, cardiotoxicity is also observed at lower doses of anthracyclines, so there is no “safe” dose below which cardiotoxicity is not a concern. 

Furthermore, LVSD due to anthracycline toxicity may occur during therapy or many years following the completion of therapy and may ultimately be irreversible if detected late [13,17]. As such, early detection of anthracycline-related LVSD may allow for some degree of functional recovery [17]. There is an increased risk for cardiotoxicity with a concurrent or sequential use of anthracyclines with other potentially cardiotoxic regimens, and/or concurrent radiation therapy. An example may be anthracycline treatment followed by HER2-targeted therapies, such as trastuzumab, for breast cancer [35,37]. 

In contrast to anthracyclines, LVSD due to HER2-targeted therapies or proteasome inhibitors alone is usually reversible and not dose-dependent [13]. HER2-targeted therapies, such as trastuzumab, are most commonly used in the treatment of HER2-positive breast cancer, which accounts for around 20% of breast cancer diagnoses. The risk of ≥10% decline in LVEF with trastuzumab has been reported to be between 9 and 18%, while the risk of symptomatic congestive heart failure (CHF) has been reported between 0.4 and 2% [38]. Carfilzomib, a proteosome inhibitor most commonly used in the treatment of multiple myeloma, is associated with a 4.4% risk of CHF based on clinical trial data [39]. Several small molecule tyrosine kinase inhibitors (TKIs) have been also associated with LVSD; however, this does not appear to be a class effect and is more likely due to off-target effects [13]. 

Immunotherapies such as immune checkpoint inhibitors (ICI), which have changed the landscape for treating cancer, have received attention for the rare, although significant, occurrence of fulminant myocarditis [40]. Though not completely understood, ICI-induced myocarditis is suggested to be related to T-cell-mediated inflammation, causing myocardial cell death [14]. Clinical presentation can include heart failure and arrhythmias or can mimic an ACS, making diagnosis complex [14]. Although ICI-mediated myocarditis is rare, occurring in approximately 1% of patients undergoing ICI therapy, in severe cases, mortality can be as high as 50% and, therefore, early and accurate identification and treatment are critical [41,42]. Emerging therapies, such as chimeric antigen receptor (CAR) T-cell therapies, may also be associated with LVSD; however, research is ongoing [43]. Most cardiac events related to CAR T-cell therapy occur in the setting of cytokine release syndrome (CRS), so it remains unclear whether CAR T-cell therapy has direct cardiotoxic effects or whether the cardiac events are attributable to cytokine release. 

Overall, it is critically important that both the treating oncologist and cardiologist involved in the ongoing care of cancer patients be aware of known and emerging associations with LVSD of various cancer therapy agents, and aggressively manage underlying cardiovascular risk factors to reduce the risk of LVSD with potentially cardiotoxic regimens. Furthermore, chemotherapeutic agents may also be associated with other forms of cardiotoxicity, including hypertension, dyslipidemia, arrhythmias, QTc prolongation, vascular disease, thrombosis, and bleeding. Although not the focus of this review, these are important cardiotoxicities for treating physicians to be familiar with. The 2022 ESC cardio-oncology guidelines are a useful reference regarding the diagnosis and management of other cardiotoxicities, which are beyond the scope of this review.

With regards to arrhythmias, atrial fibrillation (AF) and QTc prolongation (which introduces risk for torsades de pointes) are the most frequently encountered entities in patients receiving cancer therapy [44,45]. Anthracyclines, HER2-targeted therapy, and some tyrosine kinase inhibitors can be associated with QTc prolongation, and concurrent therapy with other QT prolonging medications, such as antiemetics or antibiotics, may accentuate this effect. Such patients warrant close ECG monitoring, ensuring electrolytes, particularly potassium and magnesium are replete, and a review of concurrent medications to find alternatives that may have less effect on QTc and so minimize interruption to cancer therapy [36,46,47,48]. 

AF incidence is higher in older patients, and cancer therapies are thought to interact with cardiovascular risk factors. Clinically, AF in cancer patients poses a complex clinical challenge, as thromboembolism risk is much higher in this population than the CHADSVASC2 score reflects. However, the risk of bleeding may also be significantly increased in certain cancer types [49,50]. The classic example of increased risk of AF with cancer therapy, as well as increased bleeding risk, is with the Bruton tyrosine inhibitor ibrutinib, although anthracyclines and other cancer therapies are associated with increased AF incidence. An MDT approach involving the cardiologist and oncologist is recommended [15,48]. 

## 5. Cardiac Surveillance during and after Cancer Therapy

**Figure 3:**
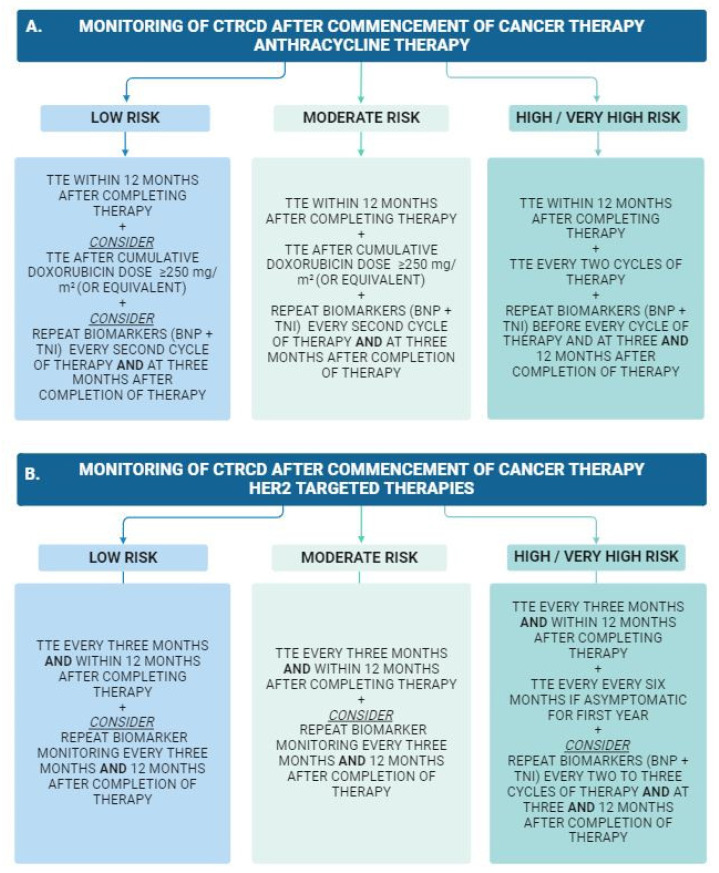
Flow chart of suggested cardiovascular monitoring for cancer patients receiving cardiotoxic cancer therapy, separated by baseline risk assessment and chemotherapeutic agent. (**A**) Anthracycline therapy. (**B**) Human epidermal growth factor receptor 2 (HER2) targeted therapies. This figure has been adapted from the 2022 ESC Guidelines on cardio-oncology [15]. TTE, transthoracic echocardiogram; BNP, brain natriuretic peptide; TnI, troponin I.

Following the baseline cardiovascular toxicity risk assessment and commencement of cancer therapy, which carries an increased risk for CTRCD, regular surveillance and monitoring are recommended to facilitate early detection of cardiovascular adverse effects. The frequency and modality of surveillance vary depending on the baseline cardiovascular toxicity risk, as well as the chemotherapeutic agent/regime, Figure 3 [15]. Furthermore, the risk of cardiotoxicity may persist after the completion of chemotherapy, particularly in patients who have received anthracyclines. A repeat echocardiogram is recommended 12 months after the completion of anthracycline therapy, even in asymptomatic patients [15]. For patients deemed to be at high cardiovascular risk, yearly clinical follow up is recommended to continue beyond this timeframe, with long-term cardiac complications of anthracycline-based chemotherapy seen up to two decades after the cessation of therapy [51]. 

### Serum Biomarkers

Cardiac biomarkers may offer an avenue for early subclinical detection of CTRCD. However, they must be interpreted within the clinical context of each patient [15]. Furthermore, the pattern of biomarker elevation varies depending on the cancer therapy type and timing of cardiotoxicity in relation to cancer therapy administration. Elevation in high-sensitivity cardiac-specific troponins, such as troponin T (TnT) and troponin I (TnI), have been suggested to be valuable for the detection of acute myocardial injury following anthracycline therapy [28]. 

Cardinale et al. reported an association between elevated TnI and reduced LVEF just one month after high-dose chemotherapy in 211 breast cancer patients [52]. Ky et al. further evaluated high sensitivity TnI, noting a correlation both with the absolute serum value and interval change from baseline following anthracycline therapy to be associated with the risk of CTRCD (HR: 1.36, 95% CI: 1.07–1.73, *p* = 0.012; HR: 1.38, 95% CI: 1.05–1.81, *p* = 0.020, respectively) [53]. Although not etiology specific, a recent meta-analysis including 2163 patients has shown elevated troponin levels following chemotherapy to be associated with an increased risk of LVSD (odds ratio [OR] 11.9, 95%CI 4.4–32.1) [54]. Furthermore, the authors reported normal troponin following treatment to have a negative predictive value of 93% for LVSD [54].

Clinically, it is important to note that elevated troponin is not specific for LVSD, and further evaluation must be conducted to characterize the underlying etiology. Specifically, an interval elevation in serum troponin may occur in ICI-related myocarditis and has been seen to correlate with poor prognosis in these patients [33,55]. Renal impairment or pulmonary pathology may also result in an elevation of troponin without any specific cardiac pathology. 

Relative changes in serum BNP or NT-proBNP have also been evaluated as a diagnostic tool for CTRCD. Sandri et al. reported an association between persistently elevated NT-proBNP and both left ventricular systolic and diastolic dysfunction following high-dose chemotherapy in 52 patients [56]. Absolute serum BNP >100 pg/mL has also been associated with an increased risk for heart failure and mortality following anthracycline therapy [57]. Pavo et al. confirmed this correlation, reporting a cutoff NT-proBNP value of >125 pg/mL to have an HR for all-cause mortality of 1.33 (95%CI 1.06–1.68, *p* < 0.013) when adjusted for age, tumor stage, cardiac status, and high-sensitivity TnT [30]. 

Overall, despite increasing evidence for the diagnostic and prognostic value of serum biomarkers for CTRCD, there are currently no widely accepted cutoff values for a biomarker-based diagnosis of CTRCD. When abnormalities in cardiac biomarkers are detected, they require further investigation with cardiac imaging, usually beginning with a transthoracic echocardiogram [15]. 

## 6. Cardiac Imaging

### Echocardiography

Echocardiography is the cornerstone for the detection, diagnosis, and surveillance of LVSD. Baseline measurement of LVEF TTE within three months prior to initiation of potentially cardiotoxic antineoplastic therapy has been recommended by multiple international consensus guidelines [2,8,12,15]. Where possible, three-dimensional (3D) TTE for LVEF and GLS, as well as right ventricular size and systolic function, is recommended within the three months preceding the commencement of potentially cardiotoxic cancer therapy [2,5,8]. If image quality does not permit 3D LVEF measurement, then Simpson’s biplane would be the next recommended quantitative approach, facilitated by the use of an ultrasound enhancing agent (referred to colloquially as “echo contrast”) if needed for endocardial border definition.

TTE is central to the diagnostic evaluation and grading of CTRCD [8,12,58]. The frequency of surveillance imaging depends on each patient’s baseline risk for cardiotoxicity. Additionally, consensus guidelines recommend a full TTE workup for any patient on cardiotoxic cancer therapy who presents with new cardiac symptoms [15]. The recently published ESC Guidelines on Cardio-oncology outline the specific frequency of TTE monitoring for chemotherapeutic drug classes based on baseline risk assessment [15]. Cutoff values for the detection of LVSD with TTE are presented in Table 1. TTE may also be clinically useful in cancer patients to detect the presence of pericardial effusion, right ventricular abnormality, or valvular disease [59]. 

**Global longitudinal strain (GLS):** GLS, often referred to as ‘strain’, as assessed by speckle tracking echocardiography (STE) on TTE, has become a well-established technique for the detection of early changes in cardiac function, often before a drop in LVEF can be detected. It is a measure of myocardial deformation [2,60]. Although strain can be measured in the longitudinal, radial, and circumferential planes of the left ventricle, it is the left ventricular GLS value that has been widely applied in the cardio-oncology setting. Multiple studies involving patients receiving chemotherapy have reported the development of heart failure to be preceded by a drop in GLS [61].

Reported to be a more sensitive marker of early systolic dysfunction than LVEF, a relative decline in GLS of 10–15% has been shown to be predictive of cardiotoxicity, with a sensitivity of 65% and a specificity of 94% [8,62,63]. A recent meta-analysis by Cocco et al. reported that a relative change in GLS from baseline is prognostic for CTRCD [64]. A relative worsening of 15% in GLS value is considered clinically relevant. Interestingly, absolute GLS with treatment was equally as valuable for the detection and prognosis of CTRCD. This supported the findings of another previous meta-analysis by Oikonomou et al. [65]. An absolute baseline value that is more negative than −18% is considered normal. However, the reference value may vary between vendors and laboratories, and some take a cutoff of −16%. Furthermore, baseline GLS pre-treatment has been shown to be predictive of CTRCD in patients receiving anthracycline therapy and is particularly useful in the setting of normal baseline LVEF [66].

**Figure 4:**
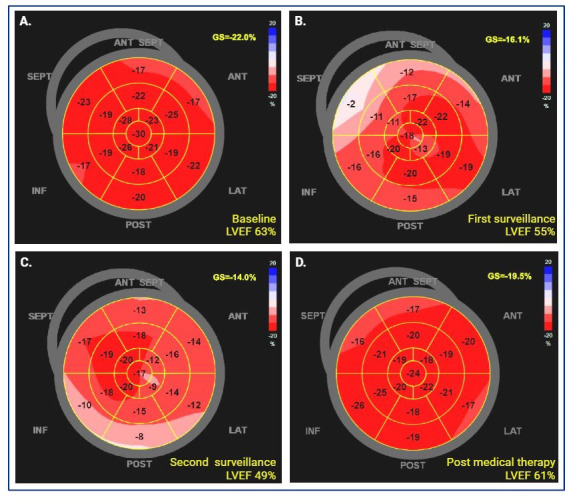
Global longitudinal strain (GLS) images of a 63-year-old female with Grade 1 invasive ductal carcinoma of the breast, estrogen receptor (ER)/progesterone receptor (PR)/human epidermal growth factor receptor-2 (HER2) receptor positive. She was treated with lumpectomy and adjuvant trastuzumab. (**A**) Baseline transthoracic echocardiogram (TTE) with normal GLS and left ventricular ejection fraction (LVEF), prior to receiving trastuzumab. (**B**) First surveillance TTE after commencement of trastuzumab therapy showed a decline in LVEF of 8% (less than the 10% cutoff for cardiotoxicity), an absolute decline in GLS of 6%, and a relative decline in GLS of 27%, consistent with mild CTRCD. (**C**) Second surveillance TTE after commencement of trastuzumab showing further reduction in LVEF to 49% and GLS to 14%. Patient was accordingly commenced on guideline-directed medical therapy (GDMT), including beta-blocker and angiotensin receptor blocker, and advised for cessation of alcohol (which she was taking in excess). No interruption in trastuzumab therapy was needed, as LVEF did not drop further with these measures. (**D**) TTE 6 months after GDMT and on completion of trastuzumab therapy with improvement in both LVEF and GLS.

Given the diagnostic and prognostic value of this technique, GLS measurement is now guideline recommended as part of the routine baseline workup and ongoing surveillance of patients undergoing potentially cardiotoxic therapy [15]. Overall, a relative decline in GLS of >15% is considered significant for CTRCD in asymptomatic patients. However, it must be noted in the context of image quality and the overall clinical picture [15]. Strain measurements may vary with different vendor packages and require high image quality and operator experience. As such, there is significant potential for inter-operator variability, and as such, it is recommended that the assessment of GLS in an individual be performed using the same vendor’s equipment and software and that adequate training be offered to ensure operator competence and quality of acquisition and interpretation [8,60]. A clinical example of GLS evaluation of CTRCD is presented in Figure 4.

**Myocardial Work:** Myocardial strain is load-dependent [67,68], and in the oncology patient population specifically, this may result in measurement variations due to volume depletion anorexia/vomiting secondary to chemotherapy administration or hypertension. As such, there has been research into the utility of myocardial work assessment on TTE, which incorporates cardiac afterload into GLS assessment and may be less affected by volume/loading conditions [67]. Currently, however, the incremental value of myocardial work assessment in the evaluation of CTRCD specifically has not yet been established [69,70,71]. 

**Diastology:** Changes in diastolic function, measured by mitral valve Doppler and tissue velocity parameters e’, E/e’ ratio, and E/A ratios, as well as left atrial size and right ventricular systolic pressure (RVSP), have been shown to precede systolic dysfunction in patients receiving chemotherapy [72]. Several small studies have reported the predictive value of early diastolic changes in the development of LVSD [73]. Despite a proposed sensitivity similar to LVEF and strain imaging, a decline in diastolic parameters is not specific to or predictive of cardiotoxicity [74]. Furthermore, variable loading conditions in this population render results inconsistent and challenging to quantify [12,73].

**Right ventricular function:** The significance and involvement of the right heart in CTRCD is not well understood and, therefore, is not currently incorporated into the guideline recommendations [8]. In a registry of patients on left ventricular mechanical support, right ventricular dysfunction was more severe and occurred more frequently in patients with chemotherapy-related cardiotoxicity when compared to other types of cardiomyopathies [12]. Furthermore, studies have suggested the involvement of right ventricular systolic dysfunction with anthracycline therapy; however, its prognostic significance is unclear [75]. Overall, guidelines suggest baseline assessment of the right ventricular size and systolic function, RVSP, and tricuspid annular plane systolic excursion (TAPSE). However, the ongoing utility of these measurements throughout cardiotoxic therapy is unclear and requires further research [12,15].

**Echocardiography and arrhythmias:** When ventricular arrhythmias arise, echocardiography is essential to assess LV systolic function as part of the evaluation. With regards to atrial fibrillation, left atrial volume index (LAVI) has been shown to be an independent associated factor for the recurrence of AF [76,77,78]. More recently, functional left atrial assessment with left atrial strain has emerged as a potential tool for predicting atrial fibrillation in the general population [79,80,81]. Although Di Lisi et al. reported left atrial strain to be a potentially useful tool for predicting subclinical CTRCD (defined as a relative drop in GLS >15%) in 169 patients with breast cancer [82], to date, however, there are otherwise no validated imaging markers to predict the risk of new-onset arrhythmia in the cardio-oncology population. 

## 7. Cardiac Magnet Resonance Imaging

Cardiac magnetic resonance imaging (CMR) is considered the gold standard in assessing both left ventricular and right ventricular volumes and function [8,12]. It is accurate and reproducible for these measurements, with high temporal and spatial resolution that may eliminate some of the limitations encountered in echocardiography [16,83]. Cardiotoxicity may be reflected on CMR as myocardial edema, necrosis, or myocardial and extracellular fibrosis [84]. Specifically, animal studies have shown anthracycline-induced cardiac changes to manifest as increased myocardial edema early following therapy, seen on CMR with T2 mapping, then subsequently as interstitial fibrosis, detected with T1 mapping and calculation of extracellular volume fraction [85]. Clinically, both myocardial T1 mapping and elevated T2 relaxation times have been suggested to be one of the earliest markers of CTRCD, predicting both subsequent myocardial fibrosis and long-term mortality in animal studies [86,87,88,89,90]. Significantly, the cessation of anthracyclines in animals with elevated T2 relation time was seen to result in the normalization of CMR findings, suggesting a role for the detection of subclinical and potentially reversible CTRCD with T2 mapping [91]. 

Overall, when compared to TTE in patients receiving anthracyclines, CMR has been found to be more accurate in detecting global and regional functional abnormalities [92]. Despite its accuracy and clinical benefits, CMR is utilized for CTRCD surveillance only when echocardiography is not available or image/window quality is non-diagnostic [5,6,15]. This is due to the limitations of CMR, which include limited access, lengthy scan time requiring breath-holding, contraindication if the patient has metal implants, and higher cost [34]. A key role of CMR in cardio-oncology is in the further evaluation of LVSD after it has been diagnosed by TTE. Myocardial characterization by CMR can help elucidate the etiology of LVSD, for which initial differentials may be broad, including infiltrative cardiomyopathies, myocarditis, and ischemic and nonischemic dilated cardiomyopathy. Additionally, an increasingly important role of CMR is in the diagnosis of ICI-related myocarditis, for which CMR findings are a major diagnostic criterion [15]. 

**Contrast and late gadolinium enhancement (LGE):** The presence and pattern of late gadolinium enhancement (LGE) on CMR provide additional value for differentiating the underlying etiology of complex cardiomyopathies due to its ability for tissue characterization and may identify myocardial edema, inflammation, and fibrosis [84]. Clinically, CMR with LGE may allow for the identification of ischemic vs. inflammatory cardiomyopathy, particularly when temporally associated with cancer therapy [27]. Specifically, LGE is particularly useful in discerning myocarditis, often ICI-mediated, from other CTRCD, typically manifesting as focal subepicardial LGE [93]. Comparatively, LGE is typically absent following anthracycline therapy, likely due to more diffuse fibrosis [86]. In patients receiving trastuzumab, LGE showed potential in detecting early myocardial changes of cardiotoxicity [94]. 

In addition to left ventricular tissue characterization in the cancer patient, CMR may have multiple other useful applications in the cancer patient. CMR is also the modality of choice for pericardial evaluation, particularly in the assessment of active pericarditis [27,95,96]. LGE can detect fibrosis and scarring, a sequelae of radiation therapy, as well as cardiotoxic chemotherapy. Scar distribution and extent may assist in diagnosis and prognostication [97]. Additionally, CMR may allow for the further characterization of cardiac masses to help distinguish thrombi from tumors. 

**Other CMR measurements:** Left ventricular mass index <57 g/m^2^ by CMR in patients receiving anthracyclines has been shown to be a predictor of adverse cardiac outcomes (sensitivity 100%; specificity 85%) [98]. CMR evaluation incorporating strain techniques may identify a decline in global circumferential strain at three months post anthracycline treatment [99]. Similarly, another study assessing patients receiving trastuzumab, CMR strain, and tissue velocity measurements detected subtle changes prior to any change in enhancement or LVEF [94]. The clinical significance and applicability of strain by CMR in clinical practice require further study. 

## 8. Nuclear Imaging

In the past, a multi-gated acquisition scan (MUGA) was the imaging modality of choice to evaluate left ventricular systolic function. This is by visualization of the cardiac blood pool via the utilization of technetium-99 and a gamma camera [100]. Strong historical data has shown its reproducibility in successive scans, establishing the foundation for its clinical use [12]. However, along with its high radiation exposure, concerns regarding the accuracy of LVEF assessment in the oncology population have been reported [101,102]. A retrospective analysis of chronic heart failure in patients receiving anthracyclines suggests it may not be as accurate in this population, where >60% of affected patients did not show a decline in LVEF [103]. Currently, MUGA is only recommended for the evaluation of CTRCD in situations where TTE is not available or non-diagnostics, and typically only where CMR is also contraindicated [5,8,15]. For the assessment of LVEF, TTE is recommended because of its ready availability, lack of radiation exposure, and ability to assess other cardiac structures simultaneously.

The primary role of nuclear imaging in the cancer patient remains testing for ischemia by way of myocardial perfusion imaging: single-photon emission computed tomography (SPECT) or positron emission tomography (PET) [8]. Although there may be a temporal association between LVSD and the initiation of chemotherapy, as with all cardiomyopathies, the first step is to exclude coronary ischemia as a cause that is a treatable etiology [15]. SPECT or PET imaging may be a useful noninvasive modality to exclude ischemia in patients who have not had an ACS or are not at high risk for coronary artery disease, thus avoiding invasive coronary angiography [27,88]. Additionally, when CMR is not available or non-diagnostic for suspected ICI-mediated myocarditis, a PET inflammation protocol study is recommended for further workup [5]. 

Nuclear imaging with technetium-99 m pyrophosphate scintigraphy (PYP) imaging also plays a role in the evaluation of cardiac transthyretin amyloidosis (ATTR), which falls under the umbrella of cardio-oncology [104,105]. In addition to serum biomarkers, a non-invasive diagnosis of cardiac ATTR can be supported when there are typical TTE/CMR findings (unexplained left ventricular wall thickening ≥ 12 mm, diastolic dysfunction, abnormal GLS on TTE with an apical sparing pattern or diffuse LGE with elevated extracellular volume on CMR). A PYP scan is consistent with cardiac amyloidosis when there is Grade 2 or 3 myocardial uptake of radiotracer and the heart-to-contralateral lung ratio is greater than or equal to 1.5 [106]. 

## 9. Cardiac Computed Tomography (CT)

The main role of cardiac CT and CT angiography in the cancer patient is its value for the assessment of the etiology of LVSD, with a high negative predictive value for the exclusion of obstructive coronary artery disease (CAD) [88,107]. It has been specifically shown to be a useful rule-out test for CAD in patients with cancer who present with suspected stress cardiomyopathy or 5-FU-related coronary vasospasm [108]. The main limitation of coronary CT is the presence of excessive calcium, which may limit the quantification of the degree of coronary stenosis. In particular, patients above the age of 70 years, those with advanced kidney disease, or those who have had previous radiation exposure to the chest may have extensive calcification that may limit the diagnostic value of this test. Renal impairment (with risk for contrast nephropathy) and tachycardia (which may degrade image quality) are also potential contraindications for this test. CT angiography is the recommended non-invasive modality for the assessment of stable chest pain in cancer patients with no previous history of coronary artery disease [108].

Recent guidelines also suggest the use of non-contrast cardiac CT for coronary artery calcium scoring as part of the workup for CVD risk assessment for all cancer patients or cancer survivors [108]. Semi-quantitative assessment may be opportunistically conducted on any chest CT or positron emission tomography (PET) completed for the evaluation of cancer. This may assist in guiding the prescription of preventative therapies [88,108]. A calcium score of more than 100 would help with risk factor modification by indicating the initiation of a statin [109]. Very high calcium scores may merit further evaluation, even in asymptomatic patients with a stress test to exclude significant ischemia. 

### Artificial Intelligence (AI)

The ever-evolving field of AI offers promise for increased efficiency, accuracy, and accessibility in the evaluation of CTRCD [110]. In the general population, many AI and machine learning (ML) models have been developed and validated to predict the risk of cardiovascular outcomes, including systolic and diastolic dysfunction, cardiac amyloidosis, significant valvular heart disease, and arrhythmias [105,111,112,113]. 

Several studies have evaluated the utility of AI and ML in the cardio-oncology population. However, further work is still needed for this specific population [114,115]. In 2020, Zhou et al. reported on the predictive power of a multivariable ML model in the prediction of CTRCD in 4300 oncology patients [116]. This group found a combination of TTE measurements with laboratory tests to have good accuracy in predicting cardiovascular outcomes, including de novo CTRCD (with AUC 0.802 in the training cohort and AUC 0.791 in the testing cohort). Li et al. also reported good accuracy (AUC 0.816) of an extreme gradient boosting ML model to predict the risk of cardiotoxicity within 30 days of fluoropyrimidine-based chemotherapy in over 36,000 colorectal cancer patients [117]. Prediction of CTRCD on baseline ECGs has also been suggested by Yai et al., who reported an increased risk of CTRCD in patients with a high risk of decreased LVEF on pre-chemotherapy ECG (adjusted HR 2.57, 95% CI 1.62–4.10, *p* < 0.001) [118]. Despite significant promise in this area, many of these models still require further large cohort external validation and refinement.

## 10. Prevention and Management of Left Ventricular Cardiotoxicity

### 10.1. Prevention

Ongoing improvements and approaches to cancer treatments aim to minimize cardiotoxicity. Considerations include, where possible, selecting cancer therapies that may have lower cardiotoxic risk while not degrading cancer outcomes, reducing doses of agents that are known to cause LVSD, and techniques, such as continuous infusion (compared to bolus administration), or lower risk formulations, such as a liposomal analog of doxorubicin [6]. If the risks and benefits of cancer treatment have been weighed, and potentially cardiotoxic cancer therapy is needed, then identification and optimizing management of the underlying cardiac disease, aggressive cardiovascular risk factor modification, and appropriate surveillance after the initiation of therapy are essential for reducing the risk of CTRCD and identifying it early when it occurs. 

There have been significant research efforts to investigate cardioprotective strategies in patients about to receive potentially cardiotoxic chemotherapy. However, the current data for efficacy are modest in most cases [119]. Non-pharmacologically, aggressive cardiovascular risk factor modification is recommended for all patients receiving chemotherapy, where possible [9,15]. This includes smoking cessation, restriction of alcohol intake, weight loss in the setting of obesity, and increased physical activity [9]; the latter two may be challenging in the patient with cancer undergoing intensive cancer therapy. Significantly, improvements in cardiopulmonary fitness may improve clinical outcomes for both active cancer patients and cancer survivors [120,121]. Furthermore, studies have shown that exercise may potentially reduce the risk of CTRCD [122,123]. Guidelines also support aggressive pharmacological management of cardiovascular risk factors in all patients, including hypertension, diabetes, and hyperlipidemia [9,15,24]. 

Furthermore, primary prevention is recommended in the guidelines for patients deemed at high or very high cardiovascular risk prior to the commencement of potentially cardiotoxic cancer therapy [15]. A meta-analysis by Caspani et al. demonstrated that cardioprotective therapies, including beta-blockers, angiotensin receptor blockers, and angiotensin converting enzyme inhibitors, were preventative of anthracycline-induced LVSD, as measured by LVEF [124]. However, there was no significant improvement in heart failure symptoms with these medications. Another meta-analysis by Li et al. confirmed these findings, extending the cardioprotective drug classes to include aldosterone antagonists (spironolactone) and [125]. They found angiotensin II receptor blockers (ARBs) to have no significant protective impact on LVEF [125]. Statin therapy has also been suggested to mitigate the risk of LVSD with anthracycline therapy in a meta-analysis. However, the results from several randomized control trials have yielded variable results [126,127,128]. The current guidelines recommend primary preventative statin therapy only in high-risk patients [15]. 

Given conflicting data for cardioprotective therapy with beta-blockers, angiotensin-converting-enzyme inhibitors (ACEi), and statins for the primary prevention of CTRCD, they are considered only on a case-by-case basis for higher risk patients and not commenced across the board. Forthcoming prospective trials in this regard may offer more clarity in the future. An imaging guided strategy with impaired GLS to commence beta blockers and ACEi for the prevention of CTRCD, as assessed in the SUCCOUR trial, found no significant difference in LVEF or GLS between groups (LVEF-directed or GLS-directed cardioprotective therapy) at longer duration follow up [129]. Nevertheless, GLS is still recommended for the early detection of CTRCD. 

Patients with pre-existing cardiovascular disease are recommended to continue on optimal guideline directed medical therapy and undergo regular monitoring, including physical examination, cardiac imaging, and biomarkers [15]. Recommendations for frequency and modality of surveillance are dependent upon the chemotherapeutic agent and the baseline cardiovascular risk assessment [5,15,28].

### 10.2. Management

The guidelines recommend all patients with CTRCD be referred to specialist cardio-oncology services for multidisciplinary management [15]. Given the spectrum of CTRCD manifestations, the accurate and early assessment of severity is imperative. Individualized patient evaluation and weighing risks, including progressive and symptomatic CTRCD and the impact of withholding or cessation of a specific cancer therapy, must occur early with interdisciplinary dialogue. 

Although the current guidelines specify the management of LVSD by chemotherapeutic agent/class, broad recommendations include the commencement and uptitration of systolic heart failure therapeutics (as per the guidelines for the treatment of any patient with acute or chronic heart failure) in all patients with symptomatic heart failure, with class I evidence [15,130]. Patients with asymptomatic LVSD are also recommended to be considered for heart failure therapy, including ACEi/ARB and a beta blocker with class IIa evidence, as well as other guideline directed medical therapies (GDMT) including mineral corticoid receptor antagonist and sodium-glucose transport protein 2 (SGLT2) inhibitors [15]. Significantly, even in patients with previously assumed irreversible anthracycline-induced LVSD, some reversibility has been observed, inversely related to the time to administration of GDMT [17]. 

Ongoing studies include consideration of additional medications with known prognostic benefits in the general heart failure population. A small prospective cohort study has also evaluated the benefit of a sacubitril-valsartan combination in CTRCD, showing significant improvement in LVEF at a median of 20 months follow up [131]. Further, Kim et al. have shown the promising prognostic value of a combination of low-dose angiotensin receptor-neprilysin inhibitor (ARNi) with SGLT2 inhibitor in rodents with CTRCD [132]. Abdel-Qadir observed a similar protective effect of SGLT2 inhibitors in 99 patients receiving anthracycline therapy, noting significantly reduced heart failure admissions compared to patients not receiving SGLT2 inhibitors. However, there was no significant difference in heart failure diagnosis or mortality [133]. SGLT2 inhibitors are also proposed to have intrinsic anti-cancer effects [134]. 

Patients commenced on therapeutic management for new chemotherapy-related LVSD are recommended to have regular clinical monitoring, including cardiac imaging and biomarkers, until at least 12 months following the completion of antineoplastic therapy [15]. Decisions regarding the duration of cardiac therapy for CTRCD after the completion of chemotherapy are individualized based on the type of cancer treatment the patient received and the patient’s cardiac function post-cancer treatment.

## 11. Conclusions

Increasing survivorship in cancer patients, along with rapidly expanding cancer therapy regimes including those with potential cardiotoxic effects, make the identification and surveillance of associated cardiovascular disease increasingly important in the oncology population. Accurate baseline cardiovascular risk assessment followed by aggressive risk-factor modification, is essential in all cancer patients. Thorough baseline cardiac evaluation, including TTE imaging and cardiac-specific biomarkers, plays an important role. 

Routine surveillance for CTRCD in both symptomatic and asymptomatic at-risk patients is essential for early diagnosis and treatment, with the dual goal of minimizing long-term cardiovascular disease and reducing the likelihood of interruption to cancer therapy in the event of CTRCD. Surveillance modality and frequency are dependent on each patient’s risk and cancer therapy related factors. Imaging must be accurate and consistent, and all possible differentials for cardiac injury often need to be evaluated to ensure appropriate cardiac therapy is initiated and to minimize the risk of interruption or discontinuation of cancer therapy. More recently, there has been an increasing number of consensus guidelines for the evaluation, surveillance, and management of this highly important population.

## Figures and Tables

**Table 1:** Summary of definitions and grading of chemotherapy associated LVSD.

	Grading	ESC 2022 [15]	ACC CardioOncology and Imaging Councils 2022 [8]	ICOS 2021 [14]	ESMO 2020 [7]	ASE 2014 [12]
**Symptomatic**	**Mild**	Mild heart failure symptoms		Mild heart failure symptoms	Asymptomatic	
	**Moderate**	Heart failure symptoms requiring outpatient therapy		Heart failure symptoms requiring outpatient therapy	Heart failure symptoms regardless of LVEF	
	**Severe**	Heart failure symptoms requiring hospitalization		Heart failure symptoms requiring hospitalization		
	**Very severe**	Heart failure symptoms requiring hospitalization *AND* inotropic support or mechanical circulatory support		Heart failure symptoms requiring hospitalization *AND* inotropic support or mechanical circulatory support		
**Asymptomatic**	**Mild**	Baseline normal LVEF (≥50%) New relative decline in GLS > 15% from baseline *OR* Elevated cardiac biomarkers from baseline	**Possible:** Reduction in LVEF ≥ 10% to overall LVEF 50–55% *OR* Reduction in LVEF by <10% to overall LVEF <50% *OR* Relative reduction in by GLS ≥ 15% with or without change in LVEF	Baseline normal LVEF (≥50%) New relative decline in GLS > 15% from baseline *OR* Elevated cardiac biomarkers from baseline	Baseline normal LVEF (≥50%) with decline in LVEF > 15%	Reduction in LVEF > 10% to overall LVEF < 53% *OR* Relative reduction in GLS > 15% from baseline is *suggestive* of CTRCD
	**Moderate**	Reduction in LVEF ≥ 10% from baseline (to LVEF 40–49%) *OR* Reduction in LVEF < 10% (to LVEF 40–49%) *AND* relative decline in GLS > 15% from baseline *OR* rise in cardiac biomarkers	**Definite:** Reduction in LVEF ≥ 10% to overall LVEF <50%	Reduction in LVEF ≥ 10% from baseline (to LVEF 40–49%) *OR* Reduction in LVEF < 10% (to LVEF 40–49%) *AND* relative decline in GLS > 15% from baseline *OR* rise in cardiac biomarkers	Reduction in LVEF ≥10% from baseline OR Reduction in LVEF to <50% (but ≥40%)	
	**Severe**	New decline in LVEF to <40%		New decline in LVEF to <40%	LVEF to <40%	

Abbreviations: ESC, European Society of Cardiology; ACC, American College of Cardiology; ICOS, International Cardio-Oncology Society; ESMO, European Society for Medical Oncology; ASE, American Society of Echocardiography; LVEF, left ventricular ejection fraction; GLS, global longitudinal strain; CTRCD, cancer therapeutic-related cardiac dysfunction.

**Table 2:** Baseline (patient-related) risk factors for cardiotoxicity [6,7,13,19].

**Patient Factors:** Age >60 or <10 years; Baseline cardiomyopathy—impaired left ventricular ejection fraction or heart failure; Known coronary artery disease; ≥Moderate valvular heart disease (particularly left-sided—mitral or aortic valve—disease); Pulmonary hypertension; Diabetes; Hypertension; Hyperlipidemia; Current smoker or smoking history; Family history of premature coronary artery disease; Obesity (body mass index ≥ 30); Peripheral vascular disease; Atrial fibrillation; Chronic kidney disease; Female sex, and post-menopausal status; Previous mediastinal radiation therapy; Prior chemotherapy with known cardiotoxic agents (e.g., anthracyclines); Genetics. *
**Baseline Cardiac Parameters and Biomarkers:** Abnormal ECG (prolonged QTc ≥ 480 ms, arrhythmia); Elevated baseline Troponin; Elevated in B-Natriuretic Peptide or N-Terminal Pro B-Natriuretic Peptide; Echocardiogram: ○ Abnormal left ventricular global longitudinal strain; ○ Impaired left ventricular ejection fraction < 50%; ○ Left ventricular hypertrophy; ○ Cardiac amyloidosis.

* Multiple genetic mutations have been associated with increased risk of anthracycline cardiotoxicity [21].

**Table 3:** Cancer therapy-related risk factors for cardiotoxicity [6,7,13,19,22].

**Cancer Treatment Factors:** Chemotherapy agents known to be associated with cardiotoxicity or CV toxic effects, for example: ○ Anthracyclines; ○ Human epidermal growth factor receptor-2 (HER2) targeted therapies; ○ Vascular endothelial growth factor inhibitors; ○ Proteosome inhibitors; ○ Immune checkpoint inhibitors; Dose of specific cardiotoxic chemotherapeutics [e.g., doxorubicin ≥ 250 mg/m^2^ or epirubicin ≥ 600 mg/m^2^]; Combination or sequential administration of cardiotoxic agents (e.g., anthracycline followed by trastuzumab); Planned concurrent mediastinal radiation [with proposed dose ≥ 30 Gy].

**Table 4:** Chemotherapy agents that may be associated with LVSD [7,10,13].

**Anthracyclines**	Doxorubicin Duanorubicin Epirubicin Idrarubicin Mitoxanthrone
**Alkylating Agents**	Cyclophosphamide Ifosfamide Mitomycin
**HER2 ^a^ targeted therapies**	Trastuzumab/Pertuzumab Lapatanib Neratinib
**Antimetabolites**	Clofarabine 5-fluorouracil Capecitabine
**Small molecule TKI**	Sunitinib ^b^ Pazopanib ^b^ Sorefenib ^b^ Dasatinib ^c^ Imatininb ^c^ Nilotinib ^c^
**MEK Inhibitors**	Trametinib
**Proteasome Inhibitors**	Carfilzomib
**Other Agents**	Interferon alpha Bevacizumab ^d^

a Human epidermal growth factor-2. b Anti-vascular endothelial growth factor (VEGF) tyrosine kinase inhibitors. c BCR-ABL tyrosine kinase inhibitors. d Anti-VEGF monoclonal antibody.
